# Cognitive impairment as a predictor of long-term psychological distress in patients with polysubstance use disorders: a prospective longitudinal cohort study

**DOI:** 10.1186/s12888-024-05600-x

**Published:** 2024-02-20

**Authors:** Jens Hetland, Astri J. Lundervold, Aleksander H. Erga

**Affiliations:** 1 Center for Alcohol and Drug Research (KORFOR), Stavanger University Hospital, P.O. Box 8100, N-4068 Stavanger, Norway; 2 Department of Biological and Medical Psychology, University of Bergen, Bergen, Norway; 3 The Norwegian Centre for Movement Disorders, Stavanger University Hospital, Stavanger, Norway; 4 Institute of Social Sciences, University of Stavanger, Stavanger, Norway

**Keywords:** MoCA, BRIEF-A, Substance use disorder, Cognitive impairment, Mental illness, Intellectual impairment

## Abstract

**Background:**

The association between polysubstance use disorder (pSUD), mental illness, and cognitive impairments is well established and linked to negative outcomes in substance use disorder treatment. However, it remains unclear whether cognitive impairment predicts long-term psychological distress among treatment seeking patients with pSUD. This study aimed to investigate the associations and predictive ability of cognitive impairment on psychological distress one and 5 years after treatment initiation.

**Methods:**

*N* = 164 treatment seeking patients with pSUD were sampled at treatment initiation. We examined associations between cognitive impairment according to Montreal Cognitive Assessment^®^ (MoCA^®^), Wechsler Abbreviated Scale of Intelligence (WASI), and Behaviour Rating Inventory of Executive Function - Adult version (BRIEF-A) administered at treatment initiation and psychological distress defined by the Symptom Check List-90-Revised (SCL-90-R) at treatment initiation, one and five years later. We ran hierarchical logistic regressions to assess the predictive ability of the respective cognitive instruments administered at treatment initiation on psychological distress measured one and five years later including psychological distress at treatment initiation and substance intake at the time-points of the measurements as covariates.

**Results:**

The main results was that MoCA^®^ and BRIEF-A predicted psychological distress at years one and five, but BRIEF-A lost predictive power when accounting for psychological distress at treatment initiation. WASI predicted psychological distress at year five, but not at year one.

**Conclusions:**

Results from MoCA^®^ and WASI was found to be less sensitive to the effect of psychological distress than BRIEF-A. Cognitive impairment at treatment initiation may hold predictive value on later psychological distress, yet its clinical utility is uncertain.

## Background

Addressing mental health is pivotal to the treatment of substance use disorders (SUDs) due to its effect on quality of life, treatment retention and risk of relapse [1–8]. Elevated psychological distress impedes individuals’ capacity to engage in long-term objectives of psychosocial improvement and moderation of substance use [9, 10] but also results in a perception of unmet treatment needs, particularly among male patients with SUDs [11]. Therefore, it is imperative to identify risk factors influencing long-term mental health to optimize the efficiency of SUD treatment.

The relationship between SUDs, mental health and cognitive functioning is intricately intertwined [12–14]. Epidemiological and clinical studies link SUD to a host of mental illnesses, such as mood and anxiety disorders, attention-deficit hyperactivity disorder, psychosis, personality disorders, suicidality and general psychological distress [13, 15–21]. Executive dysfunction, and cognitive impairments in general, are suggested to be a transdiagnostic dimension in psychopathology [22]. Indeed, psychological distress and several psychiatric disorders are associated with both specific deficits in executive function and general neurocognitive impairments, including impaired intellectual functioning [23–33]. The manifestation of cognitive impairment in apparently recovered patient cohorts implies that some cognitive impairments associated with mental illness may possess trait-like qualities [24, 34, 35].

Psychological distress and executive deficits are also considered integral transdiagnostic components of SUD and map to the withdrawal/negative affect and preoccupation/anticipation stages in the addiction cycle [12, 36–38]. Conversely, substance use may cause neuropsychological impairments [39] originating from factors such as neuroadaptations [40, 41], cerebrovascular changes [42] and hypoxia [43]. Moreover, elevated psychological distress has also been linked with cognitive impairments in patients with SUD [44–46]. This is noteworthy because cognitive impairments negatively affect several treatment processes and therapeutic change mechanisms [47–50] as well as treatment outcomes such as rates of drop-out [6, 51, 52] and relapse [53–55].

Despite the recognized link between SUD, psychological distress, and cognition, the authors are unaware of any specific research addressing the influence of cognitive functioning at the initiation of SUD treatment on long-term psychological distress in this group of patients.

### Aim

The overall objective of the current study is to evaluate the utility of administering cognitive screening instruments to inform treatment planning in a typical treatment seeking group of patients receiving treatment for polysubstance use disorder (pSUD). Clinical research on SUDs has predominantly investigated particular substances in isolation, often excluding individuals with a history of polysubstance use [56]. Nevertheless, polysubstance use is the norm in both clinical and population samples [57, 58] and represents up to 91% of treatment-seeking patients, who consume an average of 3.5 substances [59]. Additionally, individuals seeking treatment for monosubstance use disorders frequently display polysubstance use [60–65].

This study aims to 1) establish associations between cognitive impairments measured by three screening instruments at baseline (the Montreal Cognitive Assessment^®^ (MoCA^®^), Wechsler Abbreviated Scale of Intelligence (WASI) and the Behaviour Rating Inventory of Executive Function - Adult version (BRIEF-A)), and psychological distress measured according to self-reports on the Symptom Checklist 90 Revised (SCL-90-R) one and five years after enrolment in a treatment programme and 2) examine the ability of the MoCA^®^, WASI and BRIEF-A to predict psychological distress among treatment seeking in- and outpatients with pSUD receiving treatment as usual at two follow-up time points. Accordingly, we hypothesize that cognitive impairment according to at least one instrument would be associated with increased substance use and a predictor of elevated distress at follow-ups one and five years after enrolment.

## Methods

### Design

This study is part of the Stavanger Study of Trajectories of Addiction (STAYER), a prospective longitudinal cohort study of neurocognitive, psychological and social recovery in patients with SUD who initiated a new treatment sequence in the Stavanger University Hospital catchment area in Norway.

### Setting

Two hundred and eight patients were recruited at convenience from 10 specialized outpatient and residential SUD treatment facilities within the Stavanger University Hospital catchment area between March 2012 and January 2016. These facilities were diverse in terms of treatment approaches and target groups with regard to type and severity of comorbid psychiatric disorders, the severity of substance use, and degree of social adjustment and functioning. All recruitment sites are staffed by a multidisciplinary team and offers services that address a broad spectrum of psychosocial and medical issues related to SUDs. The eligibility criteria for treatment in specialized SUD-treatment services in Norway require patients to meet the diagnostic criteria for either F1x.1 harmful use, F1x.2 dependency syndrome, or F63.0 pathological gambling as defined by the ICD-10 [66]. Participants were approached by an on-site clinician working with the patient and asked if they were interested in participating in the study. Each participant was assigned a primary research assistant throughout the project and compensated approximately EUR 40 per assessment for their participation. Baseline assessment was performed after a minimum of two weeks of self-reported abstinence to minimize contamination from drug withdrawal and acute neurotoxic effects from psychoactive substances [67]. Abstinence was achieved either in a home setting or a specialized residential facility. Follow-up assessments were conducted after one and five years. Trained research personnel of the STAYER research group collected all data. Clinicians working with the patient were blinded to the assessment results obtained in the current study.

### Inclusion criteria

Inclusion criteria were as follows: a) patients enrolled in the treatment program to which they were admitted for at least two weeks; b) patients over 16 years of age; c) patients who met the diagnostic criteria for F1x.1 or F1x.2; and d) patients who reported polysubstance use defined as the consumption of multiple substances within the last year before inclusion.

### Measures

At baseline, demographic neurocognitive, psychological and social functioning data were collected through semistructured interviews, questionnaires, and selected cognitive tests administered to the patients. We used a preliminary version of the National Quality Register for Substance Abuse (KVARUS) [68], a semistructured interview to obtain information on the type of substance intake, initial age at use, treatment and work history, and educational, vocational, and social adjustment. Substance intake was measured at the one- and five-year follow-up assessments. Psychological distress was measured at baseline, as well as during the one- and five-year follow-up assessments.

#### The Montreal cognitive assessment

The Montreal Cognitive Assessment^®^ (MoCA^®^) is a cognitive screening tool that measures overall cognitive function by sampling behaviour across 14 performance tasks that engage multiple cognitive domains [65]. The test is scored in integers to obtain a total score between 0 and 30. We defined cognitive impairment (MoCA^®^+) at a sum score ≤ 25, where MoCA^®^ has demonstrated excellent sensitivity and acceptable specificity in identifying mild cognitive impairment [65]. A MoCA^®^ nonimpaired group (MoCA^®^-) was defined at sum-score > 25. MoCA® has proven effective in detecting mild cognitive impairment among patients with SUDs, exhibiting good test-retest reliability, good internal consistency, and sensitivity when utilizing the specified cut-off value [69–71].

#### The Wechsler abbreviated scale of intelligence

The Wechsler Abbreviated Scale of Intelligence (WASI) [72] comprises four subtests, two verbal measures of crystallized intelligence (Vocabulary and Similarities) and two nonverbal tests of fluent intelligence (Block Design and Matrix Reasoning). The subtests within the WASI correspond to the subtests found in the Wechsler Adult Intelligence Scale - Third Edition [73], although they feature different items. The full-scale IQ (FSIQ) was selected to reflect general intellectual function (“g-factor”). Cognitive impairment (WASI+) was delineated as an FSIQ < 86, thereby including participants with borderline intellectual functioning as cognitively impaired [45]. We also defined a WASI nonimpaired group (WASI-) as FSIQ ≥86.

#### The behavior rating inventory of executive function - adult version

The Behavior Rating Inventory of Executive Function - Adult version (BRIEF-A) is a self-report questionnaire to assess executive functioning in daily-life situations [74, 75]. The BRIEF-A comprises nine subscales and three composite scores. We utilized the cut-off scores, age norms and validation criteria proposed by the original authors [74]. Participants with cognitive impairment (BRIEF-A+) were identified by utilizing a standardized t score of ≥65 on the BRIEF-A Global Executive Composite (GEC) score. BRIEF-A GEC nonimpaired (BRIEF-A-) was defined as a GEC score < 65.

#### The symptom Checklist-90-revised

The Symptom Checklist-90-Revised (SCL-90-R) [76] is a 90-item self-report measure widely used in clinical practice and research. It has been validated for the assessment of psychological distress in patients with SUD [77], as well as in individuals with intellectual disabilities [78]. A five-point Likert scale ranging from 0 (not at all) to 4 (severely) is used to assess the level of distress experienced by respondents in the past 7 days. The checklist yields nine symptom dimension subscales: Somatization, Obsessive–Compulsive Disorder, Interpersonal Sensitivity, Depression, Anxiety, Hostility, Phobic Anxiety, Paranoid Ideation, and Psychoticism. The Global Severity Index (GSI) reflects the mean score of SCL-90-R for all reported symptoms and was employed to assess overall psychological distress. In accordance with Derogatis [76], we defined “caseness”, i.e., self-reported level of psychological distress that warrants further assessment, as a GSI standardized t score ≥ 63 or t score ≥ 63 on two or more symptom scales.

#### The drug use identification test

The Drug Use Identification Test (DUDIT) is a self-report screening tool used to evaluate substance consumption, substance-related behaviours, and substance-related problems [79]. The DUDIT consists of 11 items that are rated on a five-point Likert scale, ranging from “never” to “four or more times a week”. We used the four consumption items from DUDIT (DUDIT-C) to gauge substance intake [80]. As study participation mandated a period of abstinence from substances prior to baseline assessment, the DUDIT-C score was recorded as 0 at the baseline measurements.

### Statistical procedure

Assumptions of normality were evaluated by inspecting histograms and the Shapiro–Wilks test. To obtain optimal statistical power, we did not listwise exclude cases when some cognitive measures were missing or invalid. The distribution of the SCL-90-R GSI scores departed significantly from normality at baseline (*W* = 0.96, *p* < 0.001), year 1 (*W* = 0.92, *p* < 0.001) and year 5 (*W* = 0.89, *p* < 0.001). This was also true for age (*W* = 0.92, *p* < 0.001), years of education (*W* = 0.94, *p* < 0.001), years of work experience (*W* = 0.82, *p* < 0.001), substance debut age (*W* = 0.93, *p* < 0.001), years of substance use (*W* = 0.93, *p* < 0.001) and treatment attempts (*W* = 0.75, *p* < 0.001). Thus, we applied the Mann–Whitney U test to evaluate group differences pertaining to cognitive impairment according to the respective instrument. In accordance with Fritz et al. [81], we calculated effect sizes for these analyses. The chi-squared test of independence was used to analyse group differences for the categorical variables caseness, gender, income from work or other meaningful daily activity and intravenous drug ever used.

Additional analyses were conducted on all baseline variables to examine differences in attrition over the five-year study duration. Disparities in attrition rate based on baseline measurements of MoCA^®^, WASI and BRIEF-A has been reported elsewhere [82].

We ran hierarchal logistic regression analyses with SCL-90-R caseness as outcome at years one and five. The model was developed in three stages to evaluate the utility of administering cognitive screening instruments. Cognitive status, measured by the cognitive screening tools (MoCA^®^, WASI or BRIEF-A), was therefore entered in the first model (Model 1). DUDIT-C score from the corresponding time point of interest was added in Model 2, and baseline SCL-90-R GSI score was added in the third and final model to evaluate the effect of cognitive status when their potential effects were accounted for. Nagelkerke’s *R*^*2*^ was used to measure the goodness of fit of the regression models. Variance inflation factor diagnostics, utilizing a threshold of 2.50 [83], indicated that multicollinearity among the independent variables posed no issues in the regression models. Statistics were conducted using the statistical software package SPSS version 29 (IBM Corp., released 2022).

## Results

**Fig. 1:**
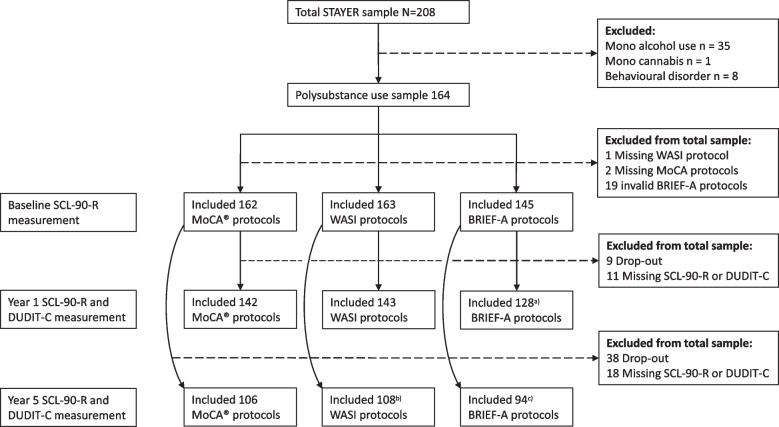
Participant inclusion, exclusion and missing data flow at baseline, 1-year, and 5-year follow-up measurements. Discrepancies between (i) excluded participants and (ii) the number of analysed protocols at baseline and follow-ups result from overlap between excluded protocols at baseline and study dropout or missing data at follow up. Specifically, a) 17 BRIEF-A protocols were excluded at the 1-year follow-up measurement, and b) 55 WASI and c) 51 BRIEF-A protocols were excluded at the 5-year follow-up measurement. MoCA^®^, Montreal Cognitive Assessment^®^; WASI, Wechsler Abbreviated Scale of Intelligence; BRIEF-A, Behaviour Rating Inventory of Executive Function – Adult version

Among the 164 participants included in this study, 145 were available for the one-year assessment, and 109 participants were available for the five-year assessment. Figure 1 presents the flow of participants and available data.

Table 1 shows the demographic characteristics of the sample at baseline, providing separate presentations of the cognitively impaired and nonimpaired groups. The BRIEF-A+ (impaired) group was younger (*Mdn* = 24.0) than the BRIEF-A- (nonimpaired) group (*Mdn* = 27.0), *U* = 5808.5, *p* = .028. *r* = .18. The BRIEF-A+ group also had more treatment attempts (*Mdn* = 1.0) than the BRIEF-A- group (*Mdn =* 1.0), *U* = 1979, *p* = .023, *r =* .19. The proportion of participants who met the criteria for caseness was approximately 76, 58 and 52% at baseline, year one and year five, respectively.

**Table 1:** Demographic features of the sample at baseline stratified according to cognitive impairment

	Total Sample	MoCA^®^ < 26		WASI FSIQ		BRIEF-A GEC	
		Impaired (*n* = 53)	Nonimpaired (*n* = 109)	Impaired (*n* = 30)	Nonimpaired (*n* = 133)	Impaired (*n* = 87)	Nonimpaired (*n* = 58)
Age at baseline, $\overline{x}$ (*SD*)	27.6 (7.5)	27.6 (7.8)	27.6 (7.4)	26.0 (8.3)	27.9 (7.3)	27.6 (8.3)*	28.7 (5.6)
Male gender, *n*(%)	107 (65.2)	35 (66.0)	71 (65.1)	18 (60.0)	88 (66.1)	60 (68.9)	36 (62.1)
Education at baseline, years, $\overline{x}$ (*SD*)	11.6 (1.7)	11.6 (1.8)	11.6 (1.7)	11.3 (1.7)	11.7 (1.7)	11.5 (1.6)	11.8 (1.8)
Income from work or other meaningful daily activity, *n*(%)	101 (61.6)	31 (58.5)	68 (62.4)	16 (53.3)	84 (63.2)	51 (58.6)	36 (62.1)
Work experience, years, $\overline{x}$ (*SD*)^a)^	5.5 (5.8)	6.4 (7.0)	5.2 (5.1)	3.9 (4.1)	5.9 (6.1)	5.5 (6.5)	6.0 (4.4)
Substance debut age, $\overline{x}$ (*SD*) ^b)^	13.1 (2.1)	13.0 (2.0)	13.2 (2.1)	12.8 (1.7)	13.1 (2.2)	13.0 (2.0)	13.5 (2.4)
Years of substance use, $\overline{x}$ (*SD*) ^b)^	14.5 (7.5)	15.0 (7.8)	14.9 (7.3)	14.9 (8.4)	14.9 (7.3)	14.7 (8.4)	15.3 (5.8)
Treatment attempts, $\overline{x}$ (*SD*)	1.6 (2.3)	1.5 (1.7)	1.8 (2.8)	1.7 (2.2)	1.7 (2.5)	1.4 (1.9)*	2.2 (3.1)
Intravenous drug ever used, *n*(%)^c)^	100 (61.3)	31 (59.6)	68 (62.4)	16 (53.3)	83 (62.9)	54 (62.8)	39 (67.2)

*MoCA*^®^ Montreal Cognitive Assessment^®^, *WASI FSIQ* Wechsler Abbreviated Scale of Intelligence Full Scale IQ, *BRIEF-A GEC* Behaviour Rating Inventory of Executive Function – Adult version Global Executive Composite, *SD* Standard Deviation. ^a)^ Missing data from 16 participants, ^b)^ Missing data from 2 participants, ^c)^ Missing data from one participant. *p < .05

### Association between cognitive impairment and SCL-90-R

Table 2 presents the SCL-90-R GSI and caseness at baseline, year one and five stratified by cognitive impairment. A significantly greater proportion classified as MoCA^®^ + (impaired) met criteria for caseness at year one χ^2^ (1, *N* = 143) = 5.63, *p* = .018, *V* = .20 and year five χ^2^ (1, *N* = 107) = 4.45, *p* = .035, *V* = .20.

**Table 2:** SCL-90-R scores stratified according to cognitive impairment assessed at baseline

Baseline	Total Sample	MoCA^®^ < 26		WASI FSIQ		BRIEF-A GEC	
		Impaired (*n* = 53)	Nonimpaired (*n* = 109)	Impaired (*n* = 30)	Nonimpaired (*n* = 133)	Impaired (*n* = 87)	Nonimpaired (*n* = 58)
SCL-90-R GSI, $\overline{x}$ (*SD*)	1.1 (0.7)	1.1 (0.7)	1.1 (0.7)	1.4 (0.8)^*^	1.1 (0.6) ^*a)^	1.4 (0.6) ^***^	0.7 (0.5) ^***^
SCL-90-R Caseness, n(%)	123 (75.9)	40 (75.5)	83 (76.5)	26 (86.7)	98 (73.7)	79 (90.8) ^***^	28 (48.3) ^***^
Year 1	Total Sample						
		Impaired (*n* = 44)	Nonimpaired (*n* = 99)	Impaired (*n* = 25)	Nonimpaired (*n* = 119)	Impaired (*n* = 76)	Nonimpaired (*n* = 53)
SCL-90-R GSI. $\overline{x}$ (*SD*)	0.8 (0.6)	1.0 (0.6)	0.8 (0.7)	0.9 (0.6)	0.8 (0.7)	1.0 (0.6) ^***^	0.6 (0.6) ^***^
SCL-90-R Caseness	84 (57.9)	32 (72.7)^*^	51 (51.5)^*^	17 (68.0)	66 (55.5)	54 (71.1) ^***^	20 (37.7) ^***^
Year 5	Total Sample						
		Impaired (*n* = 33)	Nonimpaired (*n* = 74)	Impaired (*n* = 16)	Nonimpaired (*n* = 93)	Impaired (*n* = 59)	Nonimpaired (*n* = 36)
SCL-90-R GSI, $\overline{x}$ (*SD*)	0.8 (0.7)	0.9 (0.7)	0.7 (0.6)	1.0 (0.8)	0.7 (0.7)	0.9 (0.7) **	0.5 (0.6) **
SCL-90-R Caseness, n(%)	57 (52.3)	22 (66.7)^*^	33 (44.6)^*^	13 (81.3)^*^	44 (47.3)^*^	35 (59.3) *	12 (33.3) *

*MoCA*^®^ Montreal Cognitive Assessment^®^, *WASI FSIQ* Wechsler Abbreviated Scale of Intelligence Full Scale IQ, *BRIEF-A GEC* Behaviour Rating Inventory of Executive Function – Adult version Global Executive Composite, *SCL-90-R* Symptom Checklist-90-Revised, *GSI* Global Severity Index, *SD* Standard Deviation. *P* values: *p ≤ .05 ** *p* ≤ .01 *** *p* ≤ .001

At baseline, the WASI+ (impaired) group (*Mdn* = 1.4) displayed a significantly higher SCL-90-R GSI score than the WASI- (nonimpared) group (*Mdn* = 1.0), *U* = 2480, *p* = .038, *r* = .16. Similarly, at year five, the proportion of caseness was higher for the WASI+ group than for the WASI- group χ^2^ (1, *N* = 109) = 6.30, *p* = .012, *V* = .24.

The BRIEF-A+ group was associated with all measures of SCL-90-R GSI and caseness. At baseline, the BRIEF-A+ group exhibited both a higher SCL-90-R GSI score (*Mdn* = 1.3) compared to the BRIEF-A- group (*Mdn* = 0.6), *U* = 4200, *p* < .001, *r* = .56 and a higher likelihood of caseness χ^2^ (1, *N* = 145) = 32.55, *p* < .0001, *V* = .47. At year one, the BRIEF-A+ group had significantly higher SCL-90-R GSI score (*Mdn* = 0.8) compared to the BRIEF-A- group (*Mdn* = 0.4), *U* = 2797, *p* < .001, *r* = .33 and was also associated with caseness χ^2^ (1, *N* = 129) = 14.17, *p* < .0001, *V* = .33. Similarly, at year five, the BRIEF-A+ group also had a significantly higher SCL-90-R GSI score (*Mdn* = 0.7) than the BRIEF-A- group (*Mdn* = 0.3), *U* = 1448, *p* = .003, *r* = .30. Additionally, the BRIEF-A+ group had a higher proportion of caseness compared to the BRIEF-A- group χ^2^ (1, *N* = 95) = 6.04, *p* = .014, *V* = .25.

### Differences in attrition over the five-year study duration

Participants dropping out of the study had lower education (*Mdn* = 11.0) compared to participant who did not drop out of the study (*Mdn* = 12.00), *U =* 2431, *p =* .005, *r =* .22. We did not find any baseline disparities on study drop out on the age, gender, occupational status, history with intravenous drug use, years of work, substance debut age and years of substance use.

### Prediction of SCL-90-R caseness by cognitive impairment

Tables 3 and 4 summarizes the test statistics and results obtained from the hierarchical logistic regression analyses conducted at years one and five, respectively, with the primarily aim to investigate the value of cognitive status to predict SCL-90-R caseness. At year one (Table 3), all regression models yielded a significant solution except for WASI Model 1.

**Table 3:** Summary of hierarchical logistic regression analyses at year one with SCL-90-R caseness as the dependent variable

Predictor	Model 1				Model 2				Model 3			
	*Wald*	*p*	OR	95% CI	*Wald*	*p*	OR	95% CI	*Wald*	*p*	OR	95% CI
MoCA^®^												
(Constant)	.091	.763	1.0	–	3.882	.049	.6	–	23.251	<.001	.9	–
MoCA^®^+	5.462	.**019**	2.5	1.2 – 5.4	3.388	.066	2.2	1.0–4.9	4.068	.**044**	2.7	1.0–7.0
DUDIT-C					14.415	**<.001**	1.2	1.1–1.3	11.738	**<.001**	1.2	1.1–1.3
Baseline GSI									22.393	**<.001**	6.4	3.0–13.9
	*χ2* = 5.8, *p* = .**016**, *R*^*2*^ = .054 Correctly classified 58.0%				*χ2* = 24.0, *p* =. <**.001**, *R*^*2*^ = .209 Correctly classified 70.4%				*χ2* = 54.0, *p* = **<.001**, *R*^*2*^ = .425 Correctly classified 73.9%			
WASI FSIQ												
(Constant)	1.414	.234	1.2	–	2.512	.113	.7	–	19.586	<.001	1.3	–
WASI+	1.311	.252	1.7	.7–4.3	1.144	.285	1.7	.6–4.5	.078	.780	1.2	.4–3.5
DUDIT-C					15.385	**<.001**	1.2	1.1–1.3	13.194	**<.001**	1.2	1.1–1.3
Baseline GSI									19.511	**<.001**	4.9	2.4–9.8
	*χ2 = 1.4, p = .243, R*^*2*^ *= .013* Correctly classified 57.6%				*χ2* = 20.9, *p* = **< .001**, *R*^*2*^ = .182 Correctly classified 69.2%				*χ2* = 45.6, *p* = **<.001**, *R*^*2*^ = .367 Correctly classified 73.4%			
BRIEF-A												
(Constant)	3.123	.077	.6060	–	9.650	.002	.3	–	20.707	<.001	.1	–
BRIEF-A+	13.560	**<.001**	4.1	1.9–8.6	11.124	**< 001**	3.9	1.8–8.6	.305	.581	1.3	.5–3.5
DUDIT-C					12.663	**< 001**	1.2	1.1–1,3	10.924	**<.001**	1.2	1.1–1.3
Baseline GSI									14.403	**< 001**	6.0	2.4–15.1
	*χ2* = 14.3, *p* = **< .001**, *R*^*2*^ = .141 Correctly classified 67.4%				*χ2* = 29.4, *p* = **<.001**, *R*^*2*^ = .275 Correctly classified 71.1%				*χ2* = 47.3, *p* = **<.001**, *R*^*2*^ = .414 Correctly classified 74.2%			

*MoCA*^®^ Montreal Cognitive Assessment^®^, *WASI* Wechsler Abbreviated Scale of Intelligence, *BRIEF-A* Behavior Rating Inventory of Executive Function – Adult version, *DUDIT-C* Drug Use Identification Test Consumption Items, *Baseline GSI* Baseline Symptom Checklist-90-Revised Global Severity Index, Model 1 Cognitive impairment as predictor variable, Model 2 Cognitive impairment + DUDIT-C score as predictor variables, Model 3 Cognitive impairment + DUDIT-C score + baseline SCL-90-R GSI score as predictor variables, *OR* odds ratio, *CI* confidence interval, *χ2* = Omnibus test of model coefficient, *R*^*2*^ = Nagelkerke R-squared. Significant *p* values at α = .05 in bold

**Table 4:** Summary of hierarchical logistic regression analyses at year five with SCL-90-R caseness as the dependent variable

Predictor	Model 1				Model 2				Model 3			
	*Wald*	*p*	OR	95% CI	*Wald*	*p*	OR	95% CI	*Wald*	*p*	OR	95% CI
MoCA®												
(Constant)	.861	.353	.8	–	5.470	.019	.5	–	15.258	<.001	.1	–
MoCA®+	4.336	**.037**	2.5	1.1–5.9	5.917	.**015**	3.1	1.2–7.5	5.956	.**015**	3.4	1.3–9.1
DUDIT-C					7.177	**.007**	1.1	1.0–1.3	6.180	.**013**	1.1	1.0–1.3
Baseline GSI									11.571	**<.001**	3.7	1.7–7.8
	*χ2* = 4.5, *p* = **.033**, *R*^*2*^ = .055 Correctly classified 58.9%				*χ2* = 12.7, *p* = **.002**, *R*^*2*^ = .151 Correctly classified 62.3%				*χ2* = 26.5, *p* = **<.001**, *R*^*2*^ = .295 Correctly classified 67.0%			
WASI FSIQ												
(Constant)	.269	.604	.9	–	3.492	.062	.6	–	13.658	<.001	.2	–
WASI+	5.464	.**019**	4.8	1.3–18.1	5.794	**.016**	5.2	1.4–19.8	4.322	**.038**	4.5	1.1–18.7
DUDIT-C					5.926	**.015**	1.1	1.0–1.2	4.917	**.027**	1.1	1.0–1.2
Baseline GSI									11.174	**<.001**	3.5	1.7–7.2
	*χ2* = 6.8, *p =* ***.*009***, R*^*2*^ *=* .080 Correctly classified 56.9%				*χ2* = 13.4, *p* = **.001**, *R*^*2*^ = .155 Correctly classified 65.7%				*χ2* = 26.6, *p* = **<.001**, *R*^*2*^ = .291 Correctly classified 66.7%			
BRIEF-A												
(Constant)	3.844	.050	.5	–	6.377	.012	.4	–	11.712	<.001	.2	–
BRIEF-A+	5.869	**.015**	2.9	1.2–6.9	5.436	.**020**	2.9	1.2–7.0	.634	.426	1.5	.5–4.2
DUDIT-C					3.999	**.046**	1.1	1.0–1.2	3.195	.074	1.1	1.0–1.2
Baseline GSI									11.712	.**010**	2.9	1.3–6.7
	*χ2* = 6.1, *p* = **.013**, *R*^*2*^ = .083 Correctly classified 62.1%				*χ2* = 10.0, *p* = **.007**, *R*^*2*^ = .135 Correctly classified 66.0%				*χ2* = 17.5, *p* = **<.001**, *R*^*2*^ = .226 Correctly classified 66.0%			

*MoCA*^®^ Montreal Cognitive Assessment^®^, *WASI* Wechsler Abbreviated Scale of Intelligence, *BRIEF-A* Behavior Rating Inventory of Executive Function – Adult version, *DUDIT-C* Drug Use Identification Test Consumption Items, *Baseline GSI* Baseline Symptom Checklist-90-Revised Global Severity Index, Model 1 Cognitive impairment as predictor variable, Model 2 Cognitive impairment + DUDIT-C score as predictor variables, Model 3 Cognitive impairment + DUDIT-C score + baseline SCL-90-R GSI score as predictor variables, *OR* odds ratio, *CI* confidence interval, *χ2* = Omnibus test of model coefficient; *R*^2^ = Nagelkerke R-squared. Significant *p* values at α = .05 in bold

Nagelkerke *R*^*2*^ increased from Model 1 (cognitive instrument as predictor) to Model 2 (cognitive instrument + DUDIT-C as predictor) and from Model 2 to Model 3 (cognitive instrument + DUDIT-C + Baseline GSI as predictor) across all the hierarchical regression analyses. The Nagelkerke *R*^*2*^ for the significant model solutions were in the range of .054–.425 at year one and .055–.295 at year five.

MoCA^®^ + emerged as a significant independent predictor of long-term caseness in all models, except for Model 2 at year one, where it approached significance at an α = .05 level (*p* = .066). Its odds ratios (ORs) ranged from 2.2 to 3.4. While WASI+ did not prove to be a significant predictor of caseness in the year one regression models, it gained significance in all models at year five, with ORs ranging from 4.5 to 5.2. BRIEF-A+ exhibited significant predictive ability for caseness in Model 1 and Model 2 at both year one and year five, with ORs ranging from 2.9 to 4.1. However, the statistical significance of BRIEF-A+ as a predictor was lost in Model 3 at both years one and five.

In addition, the DUDIT-C scores emerged as significant predictors of caseness in models 2 and 3 for both year one and year five, with ORs ranging from 1.1 to 1.2. Similarly, when baseline GSI was included in Model 3 at both time points, it also demonstrated significant predictive value, with ORs ranging from 2.9 to 6.4.

## Discussion

We established associations between three widely used cognitive screening tools and psychological distress and examined their ability to predict the occurrence of psychological distress at levels warranting psychiatric assessment one and five years following treatment initiation. The main finding in the current study was that the results from the selected cognitive screening instruments showed associations with psychological distress and predicted later caseness in all regression models. However, the patterns of associations and predictive value varied across the included cognitive tests. MoCA^®^ + was associated with and proved to be a significant independent predictor of long-term caseness at both the one- and five-year measurements. Notably, significance was sustained after the impact of baseline psychological distress was accounted for. Thus, the MoCA^®^ results may function as an independent predictor of long-term elevated psychological distress among patients with SUD. While WASI+ did not predict caseness at year-one, it was a significant predictor at the five-year follow up, even after accounting for the effect of psychological distress. BRIEF-A+ was associated with elevated psychological distress and caseness according to SCL-90-R GSI at all time points, but lost statistical significance as a predictor variable for caseness when baseline psychological distress was included in the regression model. The baseline SCL-90-R GSI and DUDIT-C scores obtained from the one- and five-year follow-ups emerged as significant predictors of caseness in the regression models, even after accounting for cognitive impairment according to the included cognitive screening instruments.

The explained variance across the regression models suggest that 1) the contribution from baseline GSI and DUDIT-C to the models explanatory power is approximately equal, 2) the regressions where baseline GSI and DUDIT-C are included produce models with a moderate to strong relationship with long-term caseness, compared to a weak relationship when they are excluded, and 3) the difference in explanatory power between MoCA^®^, WASI FSIQ and BRIEF-A GEC in models including baseline GSI and DUDI-C is limited.

While the MoCA^®^ was not specifically developed to detect cognitive impairments in patients with psychiatric illness or SUD, some subtests within the MoCA^®^ are shown to be sensitive to deficits in executive functioning [84]. Moreover, such deficits are recognized as hallmarks in both SUD [85] and other mental illnesses [22] but also “meaningfully associated” with SUD treatment outcomes [86]. Hagen et al. [44] suggest that MoCA^®^ is dissociated from concurrent psychological distress among patients with SUDs. It is noted that the sample in Hagen et al. [44] shares a significant overlap with the sample used in the current study. Others have demonstrated an association between MoCA^®^ and psychiatric comorbidities among patients with alcohol use disorder [46]. Depressive symptomatology has also been shown to negatively impact MoCA^®^ performance in a non-SUD population [87]. Moreover, a total of 79% of patients admitted to an acute psychiatric ward demonstrated cognitive impairment according to MoCA^®^, indicating that MoCA^®^ is sensitive to a wide range of mental illnesses [88]. Comorbid PTSD and SUD may also reduce the criterion-related validity of the MoCA^®^ in terms of its correspondence with the Repeatable Battery for the Assessment of Neuropsychological Status [89]. Notwithstanding, the current study suggests that MoCA^®^ assesses some cognitive domains that 1) to a limited extent are affected by psychological distress measured with SCL-90-R and 2) contribute to the prediction of long-term caseness.

The mechanism by which MoCA^®^ predicts long-term distress in the current study remains unknown. Previous studies have linked MoCA^®^-defined impairment to adverse treatment outcomes from isolated and formalized treatment settings [52, 90]. However, patients with cognitive impairments may follow different recovery pathways than patients without such impairments, where informal treatment processes and social structures may gain prominence in determining behavioural, psychosocial, emotional and vocational outcomes [50, 91]. Similarly, the link between MoCA^®^-derived cognitive impairments and psychological distress may partly be mediated by a complex interplay between treatment responsiveness and psychosocial factors [92]. SUDs and mental health problems are associated with and share social risk factors such as lack of healthy and committed social relationships, financial strain, housing insecurity or poor quality housing, poor education, unemployment and exposure to violence [93–97]. Moreover, individuals with SUD combat stigma and face barriers to social integration. These obstacles pose substantial challenges in their recovery or habilitation [98–100] and may contribute to sustaining or perpetuating mental health issues or substance use behaviour [101]. Cycles of relapse and dropouts may impede or worsen social adaptation in the short term, but the full psychological impact of poor social, vocational, and community functioning as well as social exclusion may not become evident until several years after experiencing poor response to treatment.

The relationship between WASI and psychological distress remains somewhat inconclusive. Measures of intellectual functioning have been associated with various mental illnesses [102, 103]. The current findings partially align with Hunt et al. [104], who reported that higher WASI Matrix Reasoning scores predicted a greater reduction in depressive symptomatology among patients receiving treatment for problematic alcohol use. The theoretical basis for the predictive capacity of WASI on psychological distress at the year five measurement, but not year one, is unknown. However, the predictive value of WASI and, to an extent, the MoCA^®^, on long-term psychological distress may be found in their capacity to provide measures of multiple and diverse cognitive domains [105]. A more general impairment profile, contrary to measures of more discrete cognitive domains, e.g., impulsivity or working memory, may hold greater significance in later stages of recovery. During these phases, stronger efforts are put on navigating the intricacies and challenges of work and social life than in early phases where goals are more demarked and the support network is more engaged. Within this context, cognitive impairment may increase psychological distress when interfering with the individuals’ coping with daily life demands.

The results of the current study indicated that the BRIEF-A was intimately linked to psychological distress. However, the ability of BRIEF-A GEC to predict clinical outcomes in terms of long-term psychological distress beyond measures of psychological distress at treatment onset appears limited. The association between psychological distress and elevated BRIEF-A self-reported executive impairments extends across diverse clinical and nonclinical cohorts, including veterans [106], patients with breast cancer, [107], adults with ADHD [33], patients diagnosed with mild or moderate depression [108], patients with neurological and neuropsychiatric conditions [109, 110], patients with brain tumors [111], older adults [112, 113], and controls [109]. Improved BRIEF-A results are also linked to decreased psychological distress among patients with a SUD one year following cessation [55]. Moreover, the BRIEF-A has shown questionable criterion-related validity pertaining to performance on objective tests of executive functioning [108, 113–115] and clinically relevant SUD treatment outcomes [82]. BRIEF-A may be particularly sensitive to latent executive deficits shared by SUD and psychiatric disorders, e.g., working memory impairments [14, 22, 116–122]. Conversely, the BRIEF-A may gauge self-reported functional debilitation associated with psychological distress or mental disorders among patients with SUD rather than impaired executive functioning as defined from psychometric tests.

The study’s results align with prior clinical and population-based research on the prevalence and developmental trajectories of mental illness, affirming that mental health problems among patients with SUD are substantial and that mental health problems act as a risk factor for later life mental health problems [15, 17, 123–125]. It is also noted that in accordance with the recommended cut-off scores from SCL-90-R [76], a substantial proportion (76%) of the participants reported a level of psychological distress at treatment initiation that warrants further assessment. Indeed, the level of psychological distress measured with SCL-90-R in the current study appears elevated compared to some SUD-cohorts [77, 126], but still comparable to other severe clinical SUD-profiles [127–129]. This suggest that a two-step screening-diagnostic assessment procedure may be redundant for patients with pSUD and that a comprehensive diagnostic assessment of mental illness could represent a more cost-efficient approach in treatment planning for all patients with pSUD. Unsurprisingly, the study reinforces the well-documented association between substance intake and greater levels of psychological distress [15, 17, 130].

### Strengths and limitations

The present study is one of few to investigate the long-term clinical outcomes in patients with cooccurring SUD and cognitive impairments. SUDs are recognized as enduring conditions, and data on long-term outcome measurements are of vital importance. The current study attempted to maximize ecological validity and sample heterogeneity. First, we utilized widely used and viable instruments that facilitate generalizability to clinical practice. Second, the study targets polysubstance users which is a clinically relevant and representative SUD sample [59]. Third, the cohort is highly heterogeneous and was recruited from diverse SUD clinics. Norway’s universal access to health care allows for the collection of a more comprehensive sample relative to countries where care is privatized and costly. Fourth, psychological distress represents a clinically relevant outcome measure, with clear implications for treatment planning and action.

The study dropout rate in the current cohort may be higher among participants with impaired intellectual functioning defined by the WASI than among those without [82]. This may potentially modify the sample characteristics pertaining to hitherto unknown key variables accounting for temporal disparities in the association between intellectual impairment and psychological distress. Moreover, the sample size pertaining to participants with intellectual impairments is modest and may mask true differences in psychological distress between the WASI+ and WASI- groups. The size of the WASI+ group is also modest, and fitting a regression model with three predictors exceeds the recommended number of events per variable in logistic regression analysis [131].

The results were not Bonferroni corrected and thus susceptible to type I error, i.e., the results may be spurious. However, there is little consensus on the conditions in which the results should be corrected. Due to the greater exploratory focus in the current study, an application of Bonferroni correction would also carry an inherent risk of committing Type II errors, which was undesirable [132].

The study employed screening instruments to evaluate cognitive functioning, which might have compromised the accuracy of identifying cognitive impairment. While a comprehensive neuropsychological assessment is the gold standard for determining neurocognitive functioning, it is not always feasible in clinical practice and research involving patients with SUDs due to the time-consuming nature of such assessment protocols and the extensive training required for their administration and interpretation. Consequently, clinicians and researchers commonly rely on short, easy to administer cognitive instrument to inform treatment and obtaining neurocognitive research data.

The timeframe from detoxification to assessment may be too short for some participants to measure stable neurocognitive impairment and psychological distress not influenced by long term withdrawal symptomology. While assessments of cognition and psychological distress were performed a minimum of two weeks after substance cessation, not all studies of long-term recovery have required 2-week substance abstinence [39]. In addition, the frequency of cognitive dysfunction according to MoCA^®^ and BRIEF-A found in the current cohort is comparable to results reported in previous studies in SUD populations [82]. Moreover, other studies employing the SCL-90-R have had a similar short cessation period [5, 129] or exhibited comparable degree of psychological distress [127, 128].

The current study utilized a MoCA^®^ cut-off score of ≤25 to detect cognitive impairment in accordance with previous recommendations to enhance comparisons and generalizability [65, 69, 71]. However, the frequency of PTSD symptomatology in the STAYER cohort is high [133], and others have recommended lowering the MoCA^®^ cut-off score to ≤23 to minimize the rate of false positives in SUD-PTSD populations [89].

Participants who dropped out of the study had lower education than those who remained. Furthermore, an earlier study on the current cohort has also indicated a higher study attrition rate among participants with cognitive impairments according to the WASI than among those without [82]. The uneven dropout profile could potentially introduce biases and limit the generalizability of the study’s findings. However, the authors are unaware of any research indicating that such biases could significantly impact the outcomes related to the objectives of the current study.

While the hierarchical regression model was valuable in showing the importance of cognitive status in predicting SCL-90-R caseness, the selection and ordering of the predictors, guided by the overall aim of the study, may have overlooked over relevant features. Future studies should aim to replicate and extend the findings of the current study.

## Conclusions

Identifying risk factors that undermine long term recovery is pivotal to ensure adequate treatment and post SUD-treatment support. The present study emphasized the importance of cognitive impairment and psychological distress at treatment initiation which was shown to predict elevated long-term psychological distress. Further studies should examine mediators between cognitive impairments and long-term psychological distress. Exploring such mediators could provide valuable insights into the underlying mechanisms and potential targets for interventions aimed at reducing psychological distress in individuals with cognitive impairments. In particular, little is known about the interaction between cognition and environmental factors in long term SUD-recovery. This represent a crucial research avenue to inform service providers and network on patients’ long term support needs. Research is also needed to develop clinically viable short assessment tools with established criterion-related and ecological validity. Such instruments should aim to reliably differentiate between potential psychopathology-driven cognitive impairment and cognitive deficits derived from substance-related neuroadaptations or neurotoxic effects.

BRIEF-A may be more sensitive to psychopathology-driven cognitive impairments than MoCA^®^ and WASI. Caution should be exercised when employing BRIEF-A within a clinical SUD context considering its potential limitations and biases. If utilized, it is crucial to corroborate the results with results from objective measures of executive functioning and a broader psychiatric evaluation. The utility of BRIEF-A may rather be evaluated and studied within the framework of being a viable tool assessing self-reported functional impairments associated with psychiatric conditions. Research should be conducted to explore the potential of BRIEF-A in differentiating between patients with psychiatric disorders and SUD while also determining the feasibility of identifying distinct BRIEF-A profiles.

Considering the high frequency of mental health issues in patients with polysubstance use disorders, it is imperative to investigate the cost–benefit ratio of implementing routine screening for mental disorders in individuals presenting with polysubstance use, as opposed to conducting a comprehensive diagnostic assessment for all.

## Data Availability

The data that support the findings of this study are available from the corresponding author upon reasonable request.

## Abbreviations

ADHD: Attention deficit/hyperactivity disorder
BRIEF-A: Behaviour rating inventory of executive function - adult version
CI: Confidence interval
DUDIT: The drug use identification test
DUDIT-C: The drug use identification test - consumption items
EUR: Euro
FSIQ: Full-scale IQ
GEC: Global executive composite
GSI: Global severity index
ICD-10: International classification of diseases, tenth revision
MoCA^®^: Montreal cognitive assessment®
OR: Odds ratio
pSUD: poly Substance use disorder
PTSD: Post traumatic stress disorder
SCL-90-R: Symptom check list-90-revised
SUD: Substance use disorder
STAYER: Stavanger study of trajectories of addiction
WASI: Wechsler abbreviated scale of intelligence
