# Correction: Cognitive impairment as a predictor of long-term psychological distress in patients with polysubstance use disorders: a prospective longitudinal cohort study

**DOI:** 10.1186/s12888-024-05774-4

**Published:** 2024-04-24

**Authors:** Jens Hetland, Astri J. Lundervold, Aleksander H. Erga

**Affiliations:** 1 Center for Alcohol and Drug Research (KORFOR), Stavanger University Hospital, P.O. Box 8100, Stavanger, N-4068 Norway; 2 Department of Biological and Medical Psychology, University of Bergen, Bergen, Norway; 3 The Norwegian Centre for Movement Disorders, Stavanger University Hospital, Stavanger, Norway; 4 Institute of Social Sciences, University of Stavanger, Stavanger, Norway

**Correction: BMC Psychiatry (2024) 24:143**10.1186/s12888-024-05600-x.

Following the publication of the original article [1], the authors identified that the number of years of psychological distress was incorrect. The sentence under the Results heading in the Abstract has been corrected.

The incorrect sentence is: WASI predicted psychological distress at year one, but not at year five.

The correct sentence is: WASI predicted psychological distress at year five, but not at year one.

The original article [1] has been corrected.
